# The prevalence and implications of single nucleotide polymorphisms in genes encoding the RNA polymerase of clinical isolates of *Staphylococcus aureus*

**DOI:** 10.1002/mbo3.1058

**Published:** 2020-05-17

**Authors:** Aishwarya Krishna, Bing Liu, Sharon J. Peacock, Sivaramesh Wigneshweraraj

**Affiliations:** ^1^ MRC Centre for Molecular Bacteriology and Infection Imperial College London London UK; ^2^ Department of Medicine Addenbrooke's Hospital University of Cambridge Cambridge UK; ^3^ Cambridge University Hospitals NHS Foundation Trust Cambridge UK; ^4^ Wellcome Trust Sanger Institute Cambridge UK; ^5^ London School of Hygiene and Tropical Medicine London UK

**Keywords:** RNA polymerase, single nucleotide polymorphisms, *Staphylococcus aureus*, transcription

## Abstract

Central to the regulation of bacterial gene expression is the multisubunit enzyme RNA polymerase (RNAP), which is responsible for catalyzing transcription. As all adaptive processes are underpinned by changes in gene expression, the RNAP can be considered the major mediator of any adaptive response in the bacterial cell. In bacterial pathogens, theoretically, single nucleotide polymorphisms (SNPs) in genes that encode subunits of the RNAP and associated factors could mediate adaptation and confer a selective advantage to cope with biotic and abiotic stresses. We investigated this possibility by undertaking a systematic survey of SNPs in genes encoding the RNAP and associated factors in a collection of 1,429 methicillin‐resistant *Staphylococcus aureus* (MRSA) clinical isolates. We present evidence for the existence of several, hitherto unreported, nonsynonymous SNPs in genes encoding the RNAP and associated factors of MRSA ST22 clinical isolates and propose that the acquisition of amino acid substitutions in the RNAP could represent an adaptive strategy that contributes to the pathogenic success of MRSA.

## Graphical abstract

The prevalence of single nucleotide polymorphisms in genes encoding the subunits of the RNA polymerase can have a diverse impact on the transcriptional program of clinical isolates of *Staphylococcus aureus*.

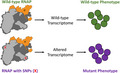

In their natural environments, bacteria must survive a multitude of stresses and cope with repeated bouts of feast and famine. The plasticity of the transcriptional program of a bacterial cell underpins its ability to adapt to and survive unfavorable growth conditions, temporally coordinate the expression of virulence genes, and tolerate exposure to abiotic and biotic stresses including antibiotics. Central to the execution and coordination of the transcriptional program in bacteria is the RNA polymerase (RNAP), which represents a nexus for the action of regulatory factors. These regulatory factors function to couple environmental and metabolic cues to control bacterial RNAP activity at a particular promoter or class of promoters. As a form of adaptive evolution, the genes encoding the subunits that make up the catalytic “core” bacterial RNAP (α, β, β′, ω; + δ and ε in Gram‐positive Firmicutes) and the σ factors, which confer promoter specificity, can acquire mutations that give rise to amino acid (aa) substitutions in response to virtually any selection pressure. These aa substitutions can affect the transcriptional program of the cell in multiple ways and thus, unsurprisingly, can lead to pleiotropic and usually advantageous phenotypic consequences. Adaptive aa substitutions due to nonsynonymous single nucleotide polymorphisms (SNPs) in genes encoding the RNAP have been previously found in long‐term evolution experiments with *Escherichia coli*. For example, Conrad et al. (2010) identified several small deletions within the gene encoding the β′ subunit of the RNAP, after the adaptation of *E. coli* strain MG1655 to growth in minimal media. More recently, studies by Rajaraman et al. (2016) and Liu et al. (2017) identified SNPs in *rpoA*, encoding the RNAP α subunit, and *rpoS*, encoding the stress response σ factor σ^S^, in *E. coli*, after adaption to growth on acetate as the sole carbon source, and acclimatization to the laboratory environment, respectively. Overall, these laboratory‐based observations suggest that the reprogramming of bacterial transcription by specific adaptive aa substitutions, that change the kinetic and regulatory performance of the RNAP, allows for optimal growth in new and challenging environments.

As the RNAP β subunit contains the binding site for the antibiotic rifampicin, adaptive aa substitutions in this subunit have been the focus of extensive study. Adaptive aa substitutions within this region, termed the rifampicin resistance determining region (RRDR), confer resistance to rifampicin in a variety of bacterial species (reviewed in Goldstein (2014)). Adaptive aa substitutions within the RRDR, that lies close to the RNAP active site, have long been recognized to elicit pleiotropic effects on gene expression (Jin & Gross, 1989). With regard to the highly versatile pathogen *Staphylococcus aureus*, Gao et al. (2013) and Baek et al. (2015) reported the identification of adaptive aa substitutions within the region encoding the RRDR that, along with conferring resistance to rifampicin, decreased susceptibility of *S. aureus* to host immune effectors and several antibiotic agents, respectively. Further, Villanueva et al. (2016) identified two SNPs within the region encoding the RRDR that enabled *S. aureus* to survive in the absence of an essential oxidative stress‐response protein Spx. Interestingly, both of these reported adaptive aa substitutions in the RRDR region have also been identified in *Salmonella enterica* and were associated with enhanced bacterial survival in aging cultures (Wrande, Roth, & Hughes, 2008).

Collectively, these reports suggest that nonsynonymous SNPs that give rise to adaptive aa substitutions often confer new properties on the bacterial transcriptional machinery, consequently eliciting pleiotropic changes in gene expression. The subsequent phenotypic alterations can contribute to the pathogenic success of bacteria, which could have further implications on both prevalence and treatment options in the clinic. However, SNPs in genes encoding the RNAP in clinical isolates of pathogenic bacteria have never been systematically analyzed. Hence, we undertook an unbiased analysis of the sequences of α, β, β′, ω, δ, and ε and σ^A^, σ^B^, σ^H^, and σ^S^ in a large collection of whole‐genome sequences of *S. aureus* isolates recovered from patients with bacteremia (Donker et al., 2017; Harris et al., 2013; Holden et al., 2013; Hsu et al., 2015; Koser et al., 2012; Reuter et al., 2016) to identify SNPs that potentially give rise to adaptive aa substitutions. The collection consisted of 1,429 MRSA isolates of sequence type 22 (ST22), a globally successful MRSA lineage, that represents both global and UK isolates. SNPs that resulted in aa substitutions were identified as described by Hsu et al. (2015) and then mapped onto a structural model of the *S. aureus* RNAP (using the *E. coli* RNAP; PDB id: 4XSY; (Bae et al., 2015) as the template) to gauge the potential implications of the aa substitutions on structure–function relationships in the RNAP.

The nucleotide sequences for the six core RNAP subunits (αββ′ωδε) and four σ factors (σ^A^, σ^B^, σ^H^, σ^S^) from 1,429 MRSA ST22 clinical isolates were aligned against the MRSA ST22 reference strain HO 5096 0412, a representative of MRSA ST22 that was isolated from a fatal neonatal infection in Suffolk, UK in February 2005 (Koser et al., 2012). Although it is possible that the entire ST22 lineage contains SNPs that are not present in other *S. aureus* lineages from other types of infection, results revealed that 144 MRSA ST22 clinical isolates (~10% of the collection) harbored at least one SNP in genes encoding the core RNAP subunits or the σ factors that were absent in the reference strain HO 5096 0412. As shown in Figure 1, 55 unique SNPs were identified across all core genes, except that encoding the ω subunit of the core RNAP. A further 25 unique SNPs were identified in genes encoding the four σ subunits. Some isolates contained SNPs in genes for both the core RNAP and σ subunits (Table 1) giving rise to RNAP variants with different combinations of aa substitutions. Thus, these variants could conceivably impact RNAP performance and the transcription program in a promoter and environment‐dependent manner. One hundred and twelve isolates (~8% of the collection) contained at least one SNP in genes encoding the major housekeeping form of the RNAP (αββ′ωδε + σ^A^). We mapped all the resulting aa substitutions on to a homology model of the structure of the *S. aureus* σ^A^‐RNAP (except for SNPs in the δ and ε subunits that were excluded from the structural model, which is based on the equivalent Gram‐negative RNAP of *E. coli* containing the σ^70^ factor where the δ and ε subunits are absent) (Figure 2). We did not identify any SNPs in the gene encoding the ω subunit, which perhaps underscores the important role the ω subunit has in the overall structural stability of the RNAP complex (Figure 1).

**FIGURE 1:**
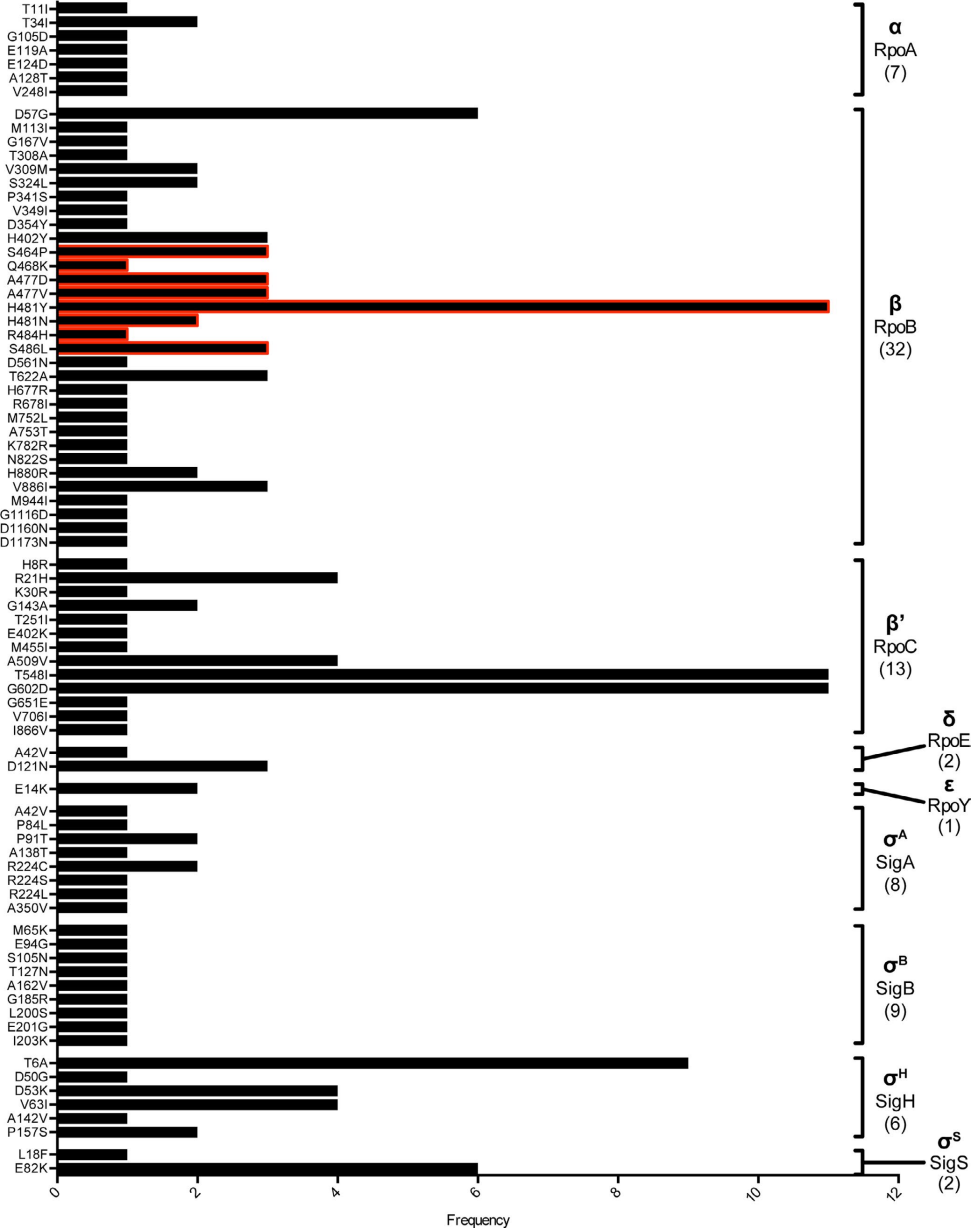
Frequency of aa substitutions in the core subunits of the *Staphylococcus aureus* RNAP (α,β,β′,δ and ε) and the 4 σ factors identified in a collection of 1,429 ST22 MRSA clinical isolates. Indicated in brackets are the number of different aa substitutions identified in each subunit. No SNPs were identified in the gene encoding the ω subunit. Highlighted in red are the eight different aa substitutions identified within the β subunit rifampicin resistance determining region (RRDR)

**TABLE 1:** List of the ST22 MRSA clinical isolates carrying more than one SNP within genes encoding the RNAP subunits and/or associated σ factors

Isolate(s)	SNP(s) in respective subunits			
	α (RpoA)	β (RpoB)	β′ (RpoC)	σ‐factor
EOE114, EOE123, EOE261		**A477V**, T622A		
601347, 601203		V309M, S324L		
BSAC3095, BSAC2299		H402Y		T6A (σ^H^)
BSAC674		V349I	A509V	E201G (σ^B^)
BSAC3129	T34I	**H481Y**		
BSAC849		**A477D**, H880R		
BSAC1447		**R484H**, H880R		
BSAC2572		**S464P**	Q402K	
BSAC598		**S464P**	T548I	
EOE113		**S486L**	G651E	
300220		D1160N	G602D	
BSAC1346			T548I	R224C (σ^A^)
ASARM121		T308A		T127N (σ^B^)
BSAC3171			K30R	E82K (σ^s^)

Note

Amino acid substitutions in the β subunit rifampicin resistance determining region (RRDR) are in bold.

**FIGURE 2:**
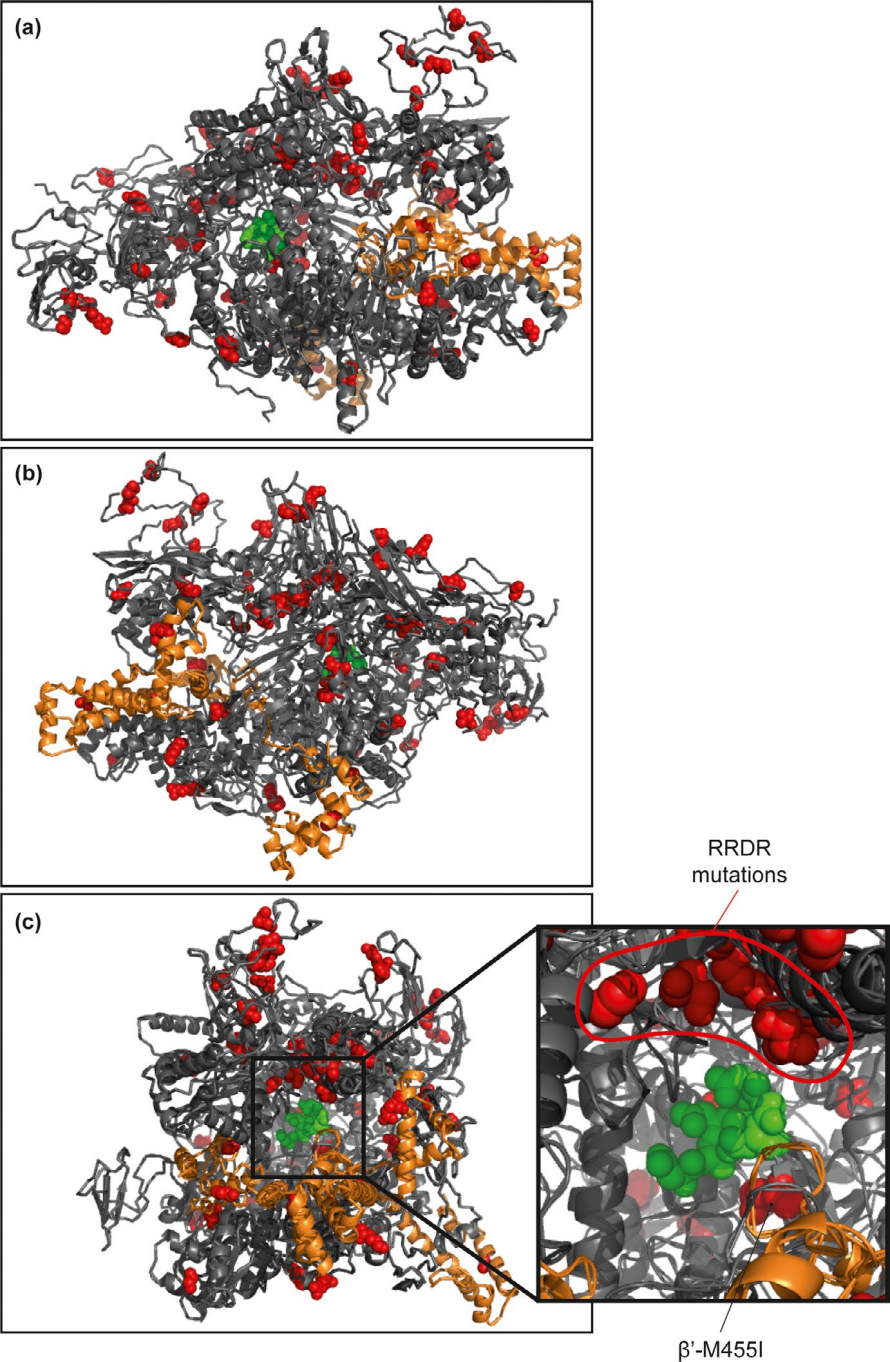
Location of aa substitutions resulting from SNPs identified in a collection of 1,429 ST22 MRSA clinical isolates mapped onto a homology model of the *Staphylococcus aureus* RNAP‐σ^A^ holoenzyme viewed from the (a) downstream and (b) upstream faces with respect to the incoming DNA (not shown) and (c) directly at the active site. Amino acid substitutions are shown in red in space‐filling mode; those identified within the active site channel are indicated in the accompanying zoomed‐in panel. The σ^A^ subunit is colored in orange and the core RNAP subunits in gray, and the conserved NADFDGD active site residues are indicated in green in space‐filling mode

Although we expected that SNPs and resulting aa substitutions would be clustered in certain regions of the RNAP, which may point to selective pressure for adaptive mutations, strikingly, no discernible “hot‐spots” for SNPs and resulting aa substitutions were noted. Only aa substitutions within the β subunit RRDR (*S. aureus* aa residues 463–550) and a single SNP, which resulted in the β′‐M455I substitution, were colocalized deep within the active site channel of the RNAP (Figure 2). The majority of SNPs in genes encoding the *S. aureus* σ^A^‐RNAP resulted in aa substitutions that appeared on the surface of the enzyme—in regions of the RNAP potentially involved in interactions with nucleic acids and/or regulatory factors (Figure 2). Hence, although we are unable to comment on how the SNPs reflect a selective pressure experienced by *S. aureus* in the host, and if so what advantage they confer, it could be that the absence of discernible “hot‐spots” for SNPs, and resulting aa substitutions, points to the inherent plasticity of the RNAP to acquire and tolerate SNPs that confer adaptive advantages to *S. aureus* during infection. Below we discuss some of the SNPs and their resulting aa substitutions in the *S. aureus* σ^A^‐RNAP and propose how they might alter the transcriptional program of the cells harboring them:

*α subunit*: A total of seven SNPs were identified in the gene encoding the α subunit (Figure 1). Six of these SNPs resulted in aa substitutions localized to the α amino‐terminal domain (α‐NTD), and a single SNP (resulting in the α‐V248I substitution) was identified in the α carboxyl‐terminal domain (α‐CTD). The α‐NTD is responsible for α subunit dimerization and forms a platform for subsequent binding of β and β′ subunits for the assembly of the core enzyme (Kimura & Ishihama, 1996; Murakami, 2015). The α‐CTD binds to specific regions upstream of the start‐site distal, −35 consensus promoter element at some promoters and can serve as a contact site for transcription regulatory factors, which stabilize the initial RNAP‐promoter complex (Browning & Busby, 2016; Murayama, Ishikawa, Chumsakul, Ogasawara, & Oshima, 2015). Although the α subunit of *E. coli* has been subjected to intensive mutagenesis analysis, none of the aa residues affected by the SNPs in our collection has been previously described. Thus, future experimental testing will reveal whether these SNPs result in aa substitutions that affect the assembly and stability of the RNAP, DNA binding, initial promoter complex formation, and interaction with transcription regulatory factors.

*β subunit*: The highest occurrence of SNPs (32) was found in the gene encoding the β subunit. Unsurprisingly, eight of the SNPs resulted in aa substitutions that fell within the RRDR and many led to known substitutions that confer resistance to rifampicin (Figure 1). The RRDR β‐H481Y substitution reported by Gao et al. (2013) occurred at the highest frequency and was present in eleven isolates (Figure 1). This aa substitution, in addition to conferring resistance to rifampicin, was reported to promote *S. aureus* immune evasion and increase the likelihood of persistent infections (Gao et al., 2013). We also noted the occurrence of the β‐A477D substitution, which was reported to decrease the susceptibility of *S. aureus* not only to rifampicin but also to vancomycin, daptomycin, and oxacillin (Baek et al., 2015). Substitution of the RRDR β‐A477 residue to valine was identified in three additional *S. aureus* isolates (Figure 1). Interestingly, this aa substitution only occurred in the presence of another β subunit substitution β‐T622A (Table 1). Whether the SNP giving rise to the β‐T622A substitution compensates for structural alterations in the RNAP induced by the RRDR β‐A477V substitution remains to be determined.

β subunit regions 1 and 2 form the “lobes” of RNAP that cover the main DNA binding channel and the DNA/RNA hybrid within the RNAP active site channel (Figure 3) (Lane & Darst, 2010). Within the β lobe‐2 region, the RNAP of *S. aureus* (and other Firmicutes) contains the lineage‐specific *Sau*βi5 insertion (corresponding to β residues 280–389) (Figure 3), in which six aa substitutions were identified. Since small deletions or single aa substitutions within or surrounding the equivalent region in the *E. coli* β subunit (*Eco*βi4) results in unstable transcription‐competent promoter complex formation (Nechaev, Chlenov, & Severinov, 2000), it is possible that the SNPs and resulting aa substitutions within *Sau*βi5 could similarly influence the *S. aureus* RNAP.

**FIGURE 3:**
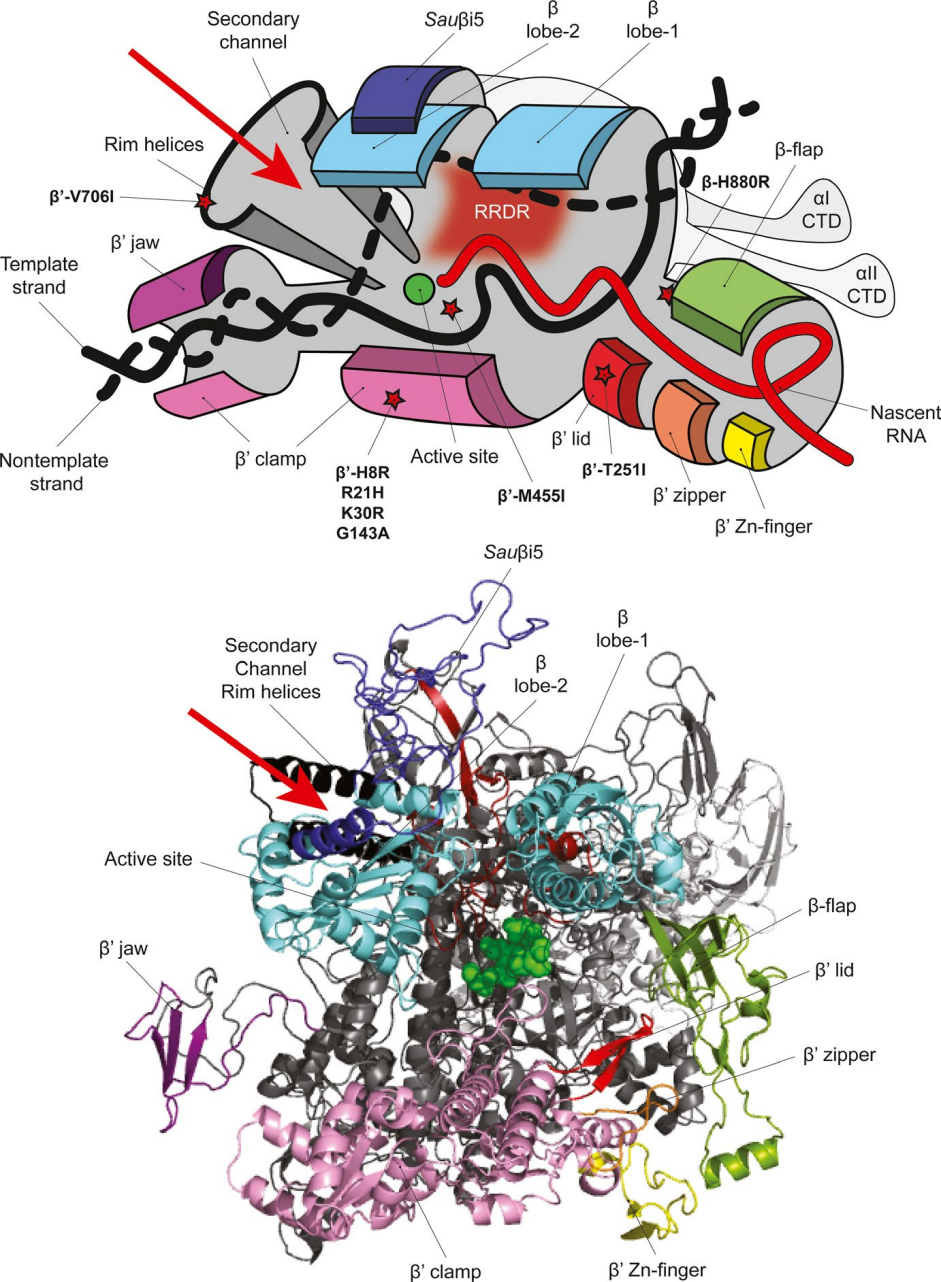
Top: schematic representation of the *Staphylococcus aureus* RNAP [inspired by Nudler (2009)] with functional domains and aa substitutions (indicated by red stars) discussed in the text labeled. Bottom: a structural model of *S. aureus* RNAP with functional domains colored and indicated as above. The RNAP α subunits are colored in light gray

The β flap is an independent structural domain that defines the RNA exit channel. It is within this channel that nascent RNA forms hairpin structures that promote transcription termination (Figure 3). Three SNPs resulting in aa substitutions (β‐N822S, β‐H880R and β‐V886I) were identified at the base of the β flap domain, which may therefore influence the transcription termination ability of the RNAP at certain promoters. Interestingly, one SNP in the β flap domain, which resulted in a conserved aa substitution β‐H880R, was only present in combination with two additional aa substitutions in the RRDR (Table 1). Based on the proximity of the β‐H880R substitution to the active site, we suspect that this aa substitution may compensate for the potentially restrictive conformational changes in the RNA exit channel introduced by the β‐A477D and β‐R484H substitutions (Figure 3).

Further, two SNPs that resulted in β‐G1116D and β‐D1160N substitutions were identified within the clamp domain of the β subunit (Figure 3). This region makes extensive interactions with the β′ subunit and, in combination with the β′ clamp, forms the “pincers” of the RNAP (Figure 3). Thus, these substitutions in the region encoding the β′clamp may alter the β–β′ interaction and conceivably the mobility of the pincers to allow productive interactions with the promoter DNA (Chakraborty et al., 2012).

*β*′* subunit*: Of the 13 SNPs identified in the gene encoding the β′ subunit, four were localized to the β′ clamp domain (Figure 3) and three of these resulted in conservative aa substitutions (β′‐H8R, R21H and K30R) and thus are unlikely to affect RNAP activity. The SNP in the β′ subunit which resulted in the β′‐T251I substitution was identified within the β′ lid domain (Figure 3) and could potentially influence the transcriptional program of the cell by affecting transcription initiation (Toulokhonov & Landick, 2006). Apart from SNPs localized to the region encoding the RRDR, only a single SNP resulting in the β′‐M455I substitution was found proximal to the RNAP active site. As this region of the RNAP interfaces with the ω subunit, which aids in the assembly of the core RNAP (Murakami, 2015), it is possible that the β′ M455I substitution may indirectly affect RNAP complex stability by affecting the β′–ω interface.

Ribonucleotide substrates gain access to the RNAP active site via a secondary channel in the β′ subunit (Figure 3); the set of helices lining the rim of this channel are referred to as the secondary channel rim helices. Only a single SNP, resulting in the β′‐V706I substitution, was identified in the rim helices, which may alter access of RNA substrates and also the interaction of transcription regulatory factors such as DksA and Gre‐factors, that are known to bind to this region (Hochschild, 2007; Laptenko, Lee, Lomakin, & Borukhov, 2003).

Interestingly, one of the most frequently identified SNPs in the gene encoding the β′ subunit was one which resulted in the β′‐G602D substitution (Figure 1), present in 11 isolates. While this SNP has been reported previously in a rifampicin‐resistant *S. aureus* isolate in combination with the β‐D471Y substitution in the RRDR (O′Neill, Huovinen, Fishwick, & Chopra, 2006)—in the collection of bacteremia isolates studied here, the SNP resulting in the β′‐G602D substitution was never associated with any mutations in the RRDR. This suggests that the β′‐G602D substitution may represent a “precursor” for the acquisition of a rifampicin resistance conferring substitution in the RRDR or may confer additional properties to the RNAP that warrant further investigation.

*σ^A^ subunit:* Most σ factors contain 4 conserved domains that are further divided into regions. Domains 2 and 4 are considered the most well conserved, with regions 2.1–2.2 interacting directly with RNAP (Nagai & Shimamoto, 1997; Paget, 2015). We identified a single SNP within region 2.1, which resulted in the σ^A^‐A138T substitution that we predict could impair the σ^A^‐RNAP interface (Figure 4), with potential ramifications on the intracellular competition for core RNAP by σ factors in response to certain cues. Regions 2.4 and 4.2 are responsible for recognition of the consensus −10 and −35 promoter elements, respectively, and region 4.2 also serves as a contact site for transcription regulatory factors (Paget, 2015; Zuo & Steitz, 2015). While no aa substitutions were identified within region 2.4, one was identified within region 4.2, resulting in the σ^A^‐A350V substitution. Since this aa position is highly conserved among primary bacterial σ factors (Lonetto, Gribskov, & Gross, 1992), it is possible that this SNP could alter recognition of consensus −35 promoter elements at a subset of promoters and/or affect the way transcription regulatory factors interact with the RNAP (Figure 4). In addition to the −10 and −35 consensus promoter elements, some bacterial promoters contain a so‐called extended −10 element, that is recognized by region 3.0 of primary σ factors (Zuo & Steitz, 2015). Three different aa substitutions were identified at residue 224 in region 3.0 of σ^A^, which resulted in the substitution of arginine to either cysteine, serine or leucine. We propose that these aa substitutions might alter the ability of the *S. aureus* σ^A^‐RNAP to effectively recognize promoters that contain an extended −10 element (Figure 4).

**FIGURE 4:**
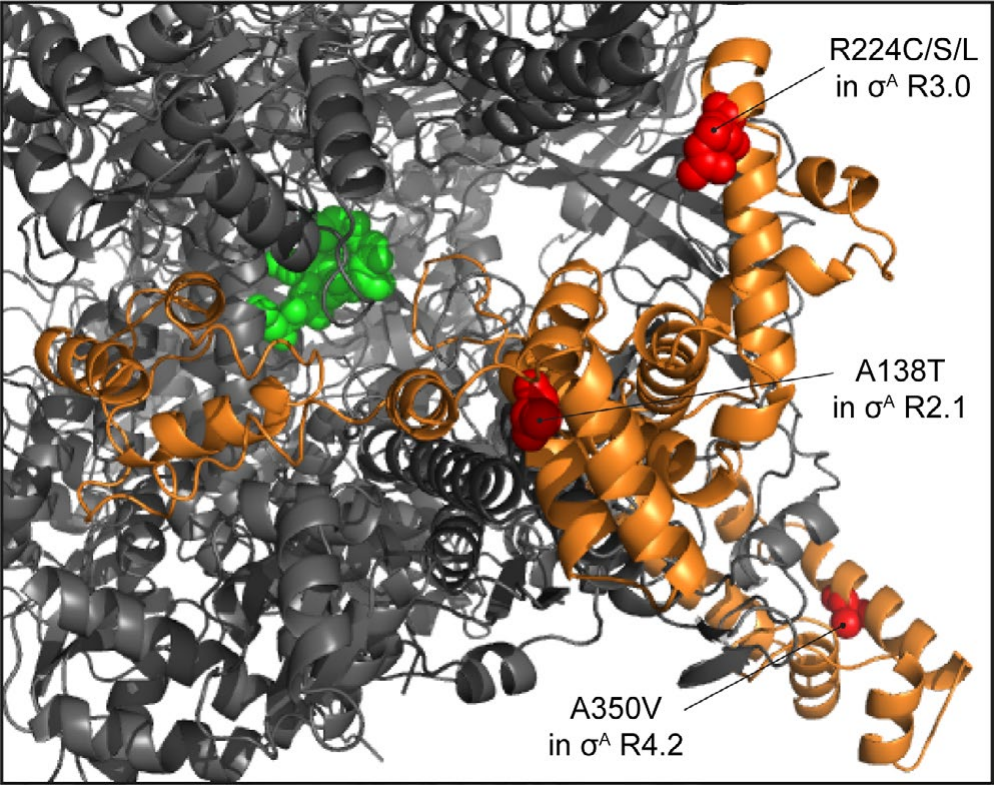
A close‐up view of the σ^A^‐RNAP interface from the homology model of the *Staphylococcus aureus* RNAP‐σ^A^ holoenzyme relating to the aa substitutions discussed in the accompanying text. The structural model is color coded as in Figure 2

In summary, perhaps the most striking observation from our analysis is the absence of any discernible hot‐spots in the RNAP at which SNPs and resulting aa substitutions accumulate. The majority of aa substitutions were scattered over/among surface‐accessible regions of the RNAP. While some aa substitutions may alter the performance of the RNAP, and hence the transcriptional program of the cell as proposed above, future work must focus on genome‐wide association analyses to search for potential aa substitutions in global regulatory factors and/or promoter regions that are associated with the SNPs in genes encoding the RNAP. For example, SNPs in the regulatory regions of some genes may compensate for the SNPs identified in the *σ* factors. We propose evaluating the impact of the nonsynonymous SNPs and resulting substitutions in the RNAP on the transcriptional program at a biochemical level, as well as in vivo under clinically relevant conditions in isogenic background strains; this could provide mechanistic insights into how the aa substitutions affect RNAP performance and consequently the virulence and antibiotic‐susceptibility profile of the *S. aureus* bacteremia isolates. Based on our analysis, we propose that SNPs in the genes encoding subunits and associated factors that make up the bacterial transcription machinery are likely to be wide‐spread in clinical bacterial isolates; deeper molecular analysis of their impact on the transcriptional program, and the phenotypic traits they confer, might provide new information on bacterial adaptive processes, the evolution of antibiotic resistance and opportunities for the development of novel diagnostic markers to evaluate bacterial infections.

## CONFLICT OF INTEREST

None declared.

## AUTHOR CONTRIBUTION

**Aishwarya** **Krishna:** Formal analysis (lead); writing – original draft (lead). **Bing Liu:** Visualization (lead). **Sharon J. Peacock:** Resources (lead); writing – original draft (supporting). **Sivaramesh Wigneshweraraj:** Conceptualization (lead); funding acquisition (lead); supervision (lead); writing – review & editing (lead).

## ETHICS STATEMENT

None required.
